# A Mouth and Tongue Interactive Device to Control Wearable Robotic Limbs in Tasks where Human Limbs Are Occupied

**DOI:** 10.3390/bios14050213

**Published:** 2024-04-24

**Authors:** Hongwei Jing, Tianjiao Zheng, Qinghua Zhang, Benshan Liu, Kerui Sun, Lele Li, Jie Zhao, Yanhe Zhu

**Affiliations:** State Key Laboratory of Robotics and System, Harbin Institute of Technology, Harbin 150001, China; 19b908070@stu.hit.edu.cn (H.J.); zhengtj@hit.edu.cn (T.Z.); 19b908069@stu.hit.edu.cn (Q.Z.); 19b908066@stu.hit.edu.cn (B.L.); 21b908043@stu.hit.edu.cn (K.S.); 23b908056@stu.hit.edu.cn (L.L.); jzhao@hit.edu.cn (J.Z.)

**Keywords:** human–robot interaction, mouth and tongue device, human augmentation, wearable robotic limbs, supernumerary robotic limbs

## Abstract

The Wearable Robotic Limb (WRL) is a type of robotic arm worn on the human body, aiming to enhance the wearer’s operational capabilities. However, proposing additional methods to control and perceive the WRL when human limbs are heavily occupied with primary tasks presents a challenge. Existing interactive methods, such as voice, gaze, and electromyography (EMG), have limitations in control precision and convenience. To address this, we have developed an interactive device that utilizes the mouth and tongue. This device is lightweight and compact, allowing wearers to achieve continuous motion and contact force control of the WRL. By using a tongue controller and mouth gas pressure sensor, wearers can control the WRL while also receiving sensitive contact feedback through changes in mouth pressure. To facilitate bidirectional interaction between the wearer and the WRL, we have devised an algorithm that divides WRL control into motion and force-position hybrid modes. In order to evaluate the performance of the device, we conducted an experiment with ten participants tasked with completing a pin-hole assembly task with the assistance of the WRL system. The results show that the device enables continuous control of the position and contact force of the WRL, with users perceiving feedback through mouth airflow resistance. However, the experiment also revealed some shortcomings of the device, including user fatigue and its impact on breathing. After experimental investigation, it was observed that fatigue levels can decrease with training. Experimental studies have revealed that fatigue levels can decrease with training. Furthermore, the limitations of the device have shown potential for improvement through structural enhancements. Overall, our mouth and tongue interactive device shows promising potential in controlling the WRL during tasks where human limbs are occupied.

## 1. Introduction

In recent years, there has been a growing focus on research related to Wearable Robotic Limbs (WRLs) [1,2,3]. This research encompasses various applications of WRLs, including affixing them to the shoulders to assist with overhead support tasks [4], wearing them around the waist to aid in daily life activities [5], mounting them on the back for carrying loads [6], and incorporating supplementary robotic digits to enhance grip strength [7] and piano playing skills [8]. Additionally, there have been studies focusing on improving the quality of life for individuals with disabilities, such as using extra robotic limbs to aid hemiplegic patients during eating [9], developing robotic arms capable of object grasping based on voice and head movements [10], and utilizing extra robotic fingers to assist individuals with disabilities in grasping objects [11]. In these applications, the primary function of WRLs is to collaborate with human users and facilitate tasks such as providing support, grasping objects, and manipulation.

The interactive interfaces and methods of WRLs present a significant research challenge. The objective is to enable users to manipulate these additional robotic limbs with the same naturalness and freedom as their own limbs. Noteworthy contributions have emerged in this field, including the coordination of WRL movements with human limbs using wearable sensors for overhead tasks [12], the control of external limbs through foot and shoulder sensors for everyday tasks [13,14], the use of eye tracking sensors for precise control of WRLs [15], the utilization of chest and abdominal muscles to control WRLs through electromyography (EMG)-based sensors [16], the classification of human intentions towards external limbs through electroencephalography (EEG) [17,18], the integration of human movement with foot EMG for task assistance [19], and shared control strategies that align human intent with robot autonomy [9,17,20]. These contributions have advanced the control of WRLs, yet challenges still remain, such as limited adaptability to different applications, potential constraints on human movement, and stability-related issues.

In industrial settings, workers often encounter complex tasks that are challenging to perform efficiently on an individual basis. WRLs can collaborate with workers to alleviate operational difficulties and enhance productivity. However, in situations where workers’ limbs are heavily engaged, novel interaction approaches are required to address the challenges of using WRLs. Industrial environments are characterized by intricate conditions and high levels of noise, making non-verbal cues, particularly body movements, more reliable for communication. Thus, utilizing the mouth and tongue for WRL manipulation is a favorable choice as it does not interfere with ongoing tasks. The mouth and tongue are naturally dexterous, being involved in speech and food manipulation. Interactive devices based on tongue control have already found applications in assistive technology for individuals with disabilities. Existing research has explored various tongue-driven interactive devices, including sensing panels placed in the upper oral cavity for mouse or robotic arm control [21,22,23], as well as internally or externally positioned magnetic induction sensors to track the spatial movements of the tongue [24,25]. However, some of these studies involve the inconvenience of attaching small magnetic induction devices to the tongue. Other investigations have focused on mouth-operated tabletop fixtures for computer screen manipulation [26], incorporating multiple inertial measurement units (IMUs) and air pressure sensors mounted on the head and within the mouth to detect manipulation intent [27] and utilizing an intraoral capsule to capture tongue movements [28]. These studies demonstrate the feasibility of mouth-operated interactive devices for conveying intentions to WRLs.

**Figure 1:**
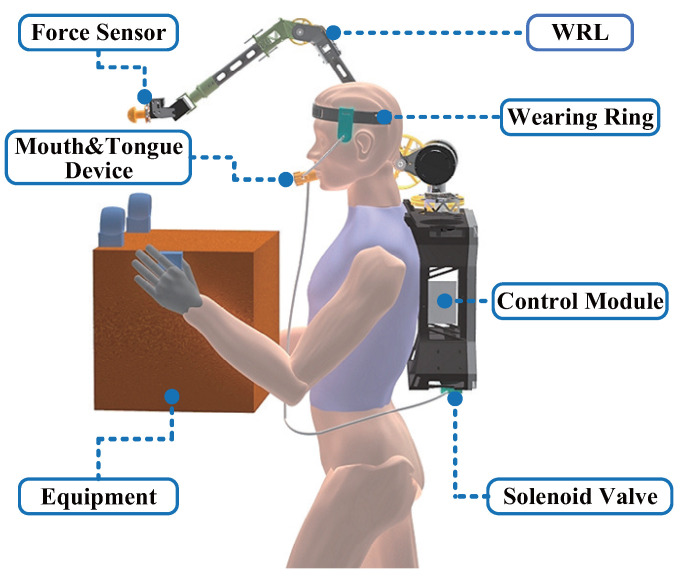
Conceptual diagram of the operator-wearable interactive system controlling the WRL.

In situations where operators’ limbs are occupied with tasks, a tongue-based interactive interface offers intuitive and flexible control. In this study, we propose the use of a mouth and tongue interactive device with feedback to assist operators in controlling the WRL. Our focus is on utilizing tongue movement and mouth gas control. By combining tongue movement and mouth gas pressure, operators can control the motion of the WRL in three dimensions and the contact force in one direction. The magnitude of the contact force can be continuously controlled using mouth gas pressure. Additionally, the mouth is sensitive to resistance sensations during exhalation and inhalation, enabling oral gas flow conditions to provide contact feedback. The WRL prototype used in this research is a six-degree-of-freedom wearable robotic arm proposed in our previous work [6]. The composition and basic concepts of the system are illustrated in Figure 1.

This paper is organized as follows: Firstly, we outline the design concept of an interactive device based on tongue movement and mouth gas pressure, providing an overview of the structure and performance of the interaction system. Secondly, methods for controlling the WRL and implementing feedback mechanisms are proposed. Specifically, a force-position hybrid control algorithm is designed for regulating contact forces in the WRL. Finally, we conduct an experiment involving ten participants who complete the pin-hole assembly task with the assistance of the WRL system to evaluate the performance of the interactive device.

## 2. Interaction Device Design

### 2.1. Design Concept and System Composition

For operators in industrial tasks, mouth movements such as speaking do not impede their ability to perform operational tasks effectively. Moreover, existing research has demonstrated the feasibility of conveying intent through interactive devices operated by the oral cavity. Therefore, wearable wireless mouth and tongue devices offer the potential to assist workers in controlling WRL. Joystick devices are particularly prevalent in industrial robot arms, gaming, and computing contexts due to their convenience and intuitive nature. We have extended this concept to tongue control, where the tongue can manipulate two in degrees of freedom: horizontally and vertically. However, achieving forward and backward movements necessitates tongue control of the joystick’s push–pull mechanism, which is difficult. To address this, a method utilizing mouth pressure for controlling the third degree of freedom in WRL is proposed. By modulating mouth gas pressure through subtle inhalation and exhalation, controlling the device’s gas pressure becomes feasible and convenient. In addition to WRL motion, interactions involving object contact also need to be considered. The WRL is equipped with a force sensor at its end, and the mouth gas pressure can be mapped to contact force, enabling one-dimensional force control. Notably, the perception of pressure changes in the mouth may be sensitive, serving as a valuable means of providing feedback concerning contact situation.

The system mainly consists of an interactive device, a host computer, and the WRL, as shown in Figure 1. The system includes an interaction module, a control module, and an execution module. The interactive module consists of a headband, mouth and tongue device, and solenoid valve. The control module comprises electronic equipment based on the Upboard controller, used to run programs and communicate. The execution module is the WRL equipped with a force sensor at the end, which can be worn on the operator’s body. Through the interactive module, the wearer can realize three-degree-of-freedom motion control, one-dimensional force control, and contact feedback of the WRL.

**Figure 2:**
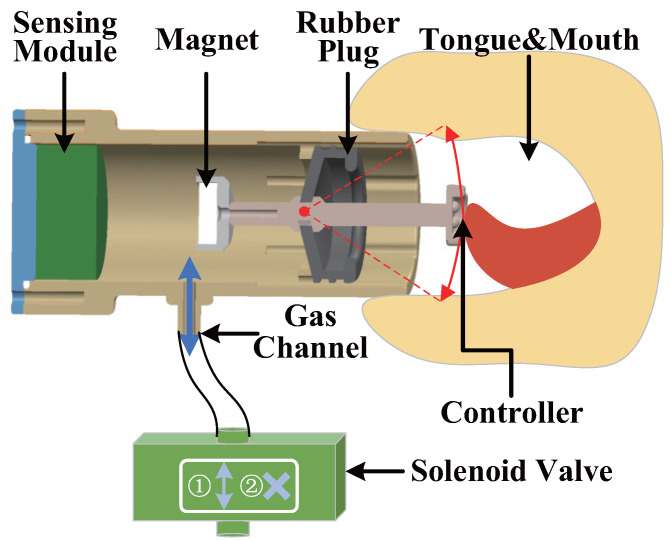
Working principle diagram of the mouth and tongue interactive device.

**Figure 3:**
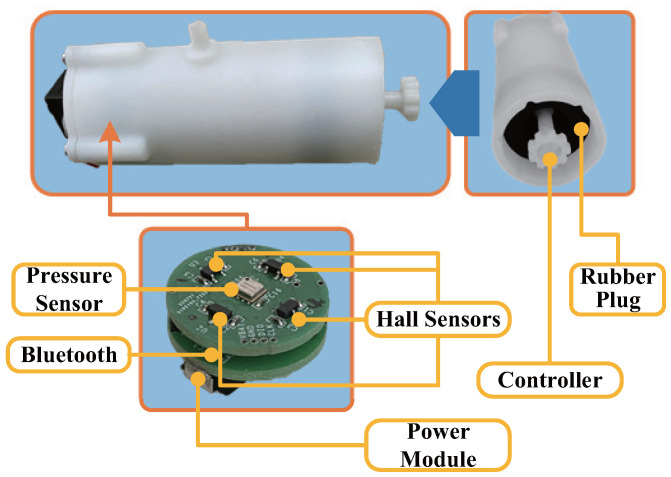
Detailed composition description of the mouth and tongue interaction device, which includes Hall sensors, a pressure sensor, Bluetooth module, a power module, a controller, and a rubber plug.

We have developed a compact mouth and tongue interactive device, primarily comprising a wireless sensing module, a magnet-tipped controller, a rubber plug, and a sealing cover, as illustrated in Figure 2 and Figure 3. Figure 2 provides an overview of the interaction device’s operational principle, while Figure 3 presents a physical representation of the device and offers a detailed breakdown of the sensing module’s components. The controller can complete a specific range of swings around the rubber plug. The elasticity of the rubber plug ensures the controller’s controllability and facilitates its automatic reset. The wireless sensing module has been custom-designed to align with our specific requirements. It incorporates four Hall sensors, a gas pressure sensor, a wireless Bluetooth module, a control module, and a power module. The power module serves to provide independent power to the wireless sensing module. The Hall and gas pressure sensors detect variations in the magnetic field and device gas pressure induced by movements at the controller’s end. The data output from the magnetic field sensor signifies variations in magnetic field intensity, measured in Gauss (Gs), whereas the data output from the gas pressure sensor denotes changes in pressure values, measured in Pascals (mbar). These datasets are encoded in a 16-bit signed-integer binary format, ensuring both the accuracy and stability of the data. The magnetic field sensor exhibits a linearity of 1.5%, while the gas pressure sensor maintains an accuracy of ±0.5 mbar. This implies that the maximum deviation between the sensor’s measured and actual values does not exceed the specified range. Subsequently, the control module processes this data and transmits it to the host computer via Bluetooth. The mouth and tongue interactive device is interconnected with the solenoid valve through a flexible tube. Inhaled and exhaled gases from the device pass through the solenoid valve passage. The host computer governs the solenoid valve channel’s opening and closing, altering the resistance of mouth gas flow, thereby furnishing feedback regarding the contact status of the WRL.

### 2.2. Data Processing and System Performance

The sensor data from the interaction device’s Hall sensors and gas pressure sensor exhibit inherent issues such as drift and noise, thereby necessitating preliminary data processing to facilitate subsequent control algorithm development. To address this, we initially apply a first-order lag filter to the sensor data. Each sensor is denoted by an index, where *i* (*i* = 1∼5) represents the sensor number. The filtering process involves the utilization of several variables. $Data_{n}^{i}$ represents the filtered data at time step n, and $Data_{n-1}^{i}$ signifies the filtering outcome at the previous time step. $Data_{raw}^{i}$ corresponds to the most recent acquired sensor data. Additionally, the filter coefficient is denoted as $B_{p}$.

(1) $$Data_{n}^{i}=\left(1-B_{p}\right)*Data_{n-1}^{i}+B_{p}*Data_{raw}^{i}$$

Data zero-point calibration and data dead zone processing are performed. In this context, $Data_{u}^{i}$ refers to the processed sensor data, $Data_{zero}^{i}$ signifies the zero-point calibration value, $Data_{zone}^{i}$ represents the dead zone setting value, and $d_{data}^{i}$ denotes the data change value.

(2) $$d_{data}^{i}=Data_{fi}^{i}-Data_{zero}^{i}$$

(3) $$Data_{u}^{i}=\left\{\begin{array}{cc} d_{data}^{i}+Data_{zone}^{i}, & \mathrm{if}\;d_{data}^{i}\leq -Data_{zone}^{i}\\ d_{data}^{i}-Data_{zone}^{i}, & \mathrm{if}\;d_{data}^{i}\geq Data_{zone}^{i}\\ 0, & \mathrm{otherwise}. \end{array}\right.$$

Subsequently, we conducted an empirical evaluation of the device’s performance. The Hall sensor demonstrated a performance range of ±100 Gs, a sensitivity of 15 mV/Gs, linearity within 1.5%, and operated at a frequency of 100 Hz. The gas pressure sensor exhibited specifications including a range from 300 to 1200 mbar, a resolution of 0.01 mbar, an accuracy of ±0.5 mbar, and a frequency of 10 Hz. The device has a total data transmission period of 100 ms, and its Bluetooth communication range extends up to 10 m, sufficiently meeting operators’ fundamental requirements. Furthermore, the solenoid valve used for feedback operates with a response frequency of 20 ms. Notably, the mouth and tongue device has a minimal mass of 31.1 g, making it exceptionally lightweight. To enhance comfort further, we designed a wearing ring weighing 118.6 g. The cumulative mass of the final wearable component totals 149.7 g, with the device’s weight distributed from the mouth to the head, effectively reducing bodily fatigue.

## 3. Methods

### 3.1. Control Strategy

To facilitate control and feedback for the WRL, an interaction method based on the acquired sensor data is devised. This control scheme, denoted as $C_{m}$, encompasses two distinct modes: the Movement Mode (MM) and the Force-Position Hybrid Mode (FM). In the MM, the operator uses the device to control the WRL’s three degrees of freedom. The FM empowers the device to regulate both the contact force and position between the WRL and the environment. The mouth and tongue interactive device governs movement instructions and contact force, with the opening and closing of the solenoid valve serving as a means of furnishing feedback information.

First, we establish the control model for MM. Leveraging the data from Hall sensors and the gas pressure sensor, a motion scheme to control the three degrees of freedom in WRL has been designed. Specifically, $Hall_{u}$, $Hall_{d}$, $Hall_{l}$, $Hall_{r}$, and $Air_{p}$ denote the processed data from the Hall sensors in the four cardinal directions and the gas pressure sensor. To capture the relationship between these sensor values and the WRL’s position, we use the proportional coefficients denoted as $\lambda_{i}$ (*i* = 1∼3). $\lambda_{1}$ is designed based on the magnitude relationship between the pressure sensor and the displacement of the robot. The angular displacement of the controller is reflected in the values of the Hall sensors, indicating the intensity of the operator’s intent for movement in this direction. $\lambda_{2}$ and $\lambda_{3}$ is set based on the magnitude of the angular displacement. Ultimately, an incremental control method is employed to calculate the positional increments at the end of the WRL, represented as $X_{d}$, $Y_{d}$, and $Z_{d}$.

(4) $$C_{m}=\left\{MM,FM\right\}$$

(5) $$\left\{\begin{array}{c} X_{d}=\lambda_{1}*Air_{p}\\ Y_{d}=\lambda_{2}*\left(Hall_{l}-Hall_{r}\right)\\ Z_{d}=\lambda_{3}*\left(Hall_{u}-Hall_{d}\right) \end{array}\right.$$

When the contact force ($F_{t}$) between the WRL and its surrounding environment surpasses the predefined threshold ($F_{m}$), a transition to the FM is initiated. This mode leverages data derived from Hall sensors to regulate the anticipated contact position ($Pos_{d}$), while gas pressure data are harnessed to govern the desired contact force ($F_{exp}$). The solenoid valve operates in one of two states: the open state ($V_{o}$) or the closed state ($V_{c}$), and this switching is denoted by the variable $V_{m}$.

(6) $$\left\{\begin{array}{c} F_{exp}=\sigma \left(Air_{p}\right)\\ Pos_{d}=\sigma \left(Hall_{l},Hall_{r},Hall_{u},Hall_{d}\right) \end{array}\right.$$

(7) $$V_{m}=\left\{V_{o},V_{c}\right\}$$

The control strategy for the interactive device is outlined in Algorithm 1. Following a successful Bluetooth connection establishment, the control mode is initially set to MM, and the solenoid valve status defaults to $V_{o}$. Both the contact force ($F_{t}$) and the gas pressure value ($Air_{p}$) are initialized to zero. The operator’s inhalation and exhalation states are discerned by comparing $Air_{p}$ with the predefined threshold value ($Air_{0}$). Subsequently, the device maps controller movements and gas pressure values to govern the WRL’s three degrees of freedom. The open solenoid valve facilitates an unobstructed airflow feedback experience for the operator’s mouth.
**Algorithm 1** Control strategies for the mouth and tongue interactive device.initial values$\;C_{m}\leftarrow NaN,V_{m}\leftarrow V_{o},M\leftarrow NaN$  Wait for the device to connect to Bluetooth  WhileTruedo  $\;\mathrm{if}\;The\;Bluetooth\;is\;connected\;\mathrm{and}\;C_{m}=NaN\;\mathrm{then}$    $\;C_{m}\leftarrow MM$    $\;F_{t}\leftarrow 0$    $\;{\mathrm{Air}}_{p}\leftarrow 0$  $\;\mathrm{if}\;C_{m}=MM\;\mathrm{and}\;F_{t}>F_{m}\;\mathrm{then}$    $\;C_{m}\leftarrow FM$    $\;V_{m}\leftarrow V_{c}$  $\;\mathrm{if}\;C_{m}=FM\;\mathrm{and}\;\left(F_{t}<F_{m}\;\mathrm{or}\;{\mathrm{Air}}_{p}<Air_{0}\right)\;\mathrm{then}$    $\;C_{m}\leftarrow MM$    $\;V_{m}\leftarrow V_{o}$  ifNo operation for along timethen    Break

The state machine continuously assesses whether the WRL is in contact with its environment, as determined by $F_{t}$. Upon contact, the mode transitions to FM, and the solenoid valve state shifts to $V_{c}$. Closing the solenoid valve provides the operator with significant feedback related to airflow resistance. In the FM, the control of X-directional motion, previously influenced by gas pressure values, transitions to force control, while YZ-directional movement control remains under the controller. In real-time, the state machine monitors the operator’s mouth gas pressure and contact force states. If the operator inhales or the WRL end becomes disengaged, the solenoid valve reopens, and the control mode reverts to MM. If the operator ceases device operation for a prolonged period, the control state is exited.

### 3.2. Force-Position Hybrid Control

When the contact force of the WRL exceeds a predefined threshold, employing MM becomes impractical, as it could potentially result in system damage and fail to meet the interaction requirements. Drawing upon an admittance control framework, an algorithm was devised to facilitate the control of force-position blending through the device. Within this mode, the X direction represents the desired force control vector, while the Y and Z directions pertain to manipulative movement axes. The operator gains control over the desired force magnitude in the X direction by modulating mouth gas pressure. Concurrently, the target force values in the Y and Z directions remain set at 0, with the option for adjustment of target positions.

To commence, we establish the foundational spring-damper model of the system and configure the system’s model parameters, denoted as *M*, *D*, and *K*. The actual and anticipated positions across the three directions are represented as $Pos_{a}$ = ${\left[Pos_{a1},Pos_{a2},Pos_{a3}\right]}^{⊤}$ and $Pos_{d}$ = ${\left[Pos_{d1},Pos_{d2},Pos_{d3}\right]}^{⊤}$, respectively. The positional deviation is expressed as $e_{pos}$ = ${\left[e_{pos1},e_{pos2},e_{pos3}\right]}^{⊤}$. The contact force $F_{t}$ = ${\left[F_{t1},F_{t2},F_{t3}\right]}^{⊤}$ is derived from the force sensor located at the extremity of the WRL. Additionally, the anticipated force is denoted as $F_{exp}$ = ${\left[F_{exp1},F_{exp2},F_{exp3}\right]}^{⊤}$, and the resultant force acting on the system is designated as $F_{te}$. The system model can be expressed as follows:

(8) $$e_{pos}=Pos_{a}-Pos_{d}$$

(9) $$M*\left({\ddot{e}}_{pos}\right)+D*\left({\dot{e}}_{pos}\right)+K*e_{pos}=F_{te}$$

The initial contact force between the WRL and the environment is denoted as $F_{0}$, which serves to sustain the system’s contact state with the environment. Additionally, $K_{f}$ represents a coefficient facilitating the correlation between air pressure and force values. The resultant force acting on the system, $F_{te}$, can be expressed as follows:

(10) $$\left\{\begin{array}{c} F_{exp1}=F_{0}+K_{f}*AirP\\ F_{exp2}=F_{exp3}=0 \end{array}\right.$$

(11) $$F_{te}=F_{t}+F_{exp}$$

$Y_{d}$ and $Z_{d}$ can be obtained by the method of movement control mode in the previous section. The desired position $Pos_{d}$ can be expressed as

(12) $$\left\{\begin{array}{c} Pos_{d1}=Pos_{a1}\\ Pos_{d2}=Pos_{a2}+Y_{d}\\ Pos_{d3}=Pos_{a3}+Z_{d} \end{array}\right.$$

By incorporating the resultant force and system deviation into the system model, where $m_{step}$ denotes the system’s time step, the terminal acceleration ($End_{acc}$) and velocity ($End_{vel}$) can be determined. Subsequently, the updated positional control variables can be represented as $Pos_{n}$ = ${\left[Pos_{n1},Pos_{n2},Pos_{n3}\right]}^{⊤}$.

(13) $$End_{acc}=M^{-1}*\left(-D*End_{vel}-K*e_{pos}+F_{te}\right)$$

(14) $$End_{vel}=End_{vel}+End_{acc}*m_{step}$$

(15) $$Pos_{n}=Pos_{a}+End_{vel}*m_{step}$$

The updated positional control parameters are integrated into the control system, enabling the implementation of the force-position hybrid control mode. When coupled with the control strategy detailed in the preceding section, this algorithm facilitates seamless transitions between FM and MM. Such an algorithm serves to enhance the system’s adaptability to varying environmental conditions.

## 4. Experiment

### 4.1. Experimental Design

The pin-hole assembly task is a typical installation task in the industry. Typically, one person holds the equipment, while another is responsible for completing the pin-hole installation, while the WRL can assist the operator in completing the task independently, the operator’s limbs are highly engaged. Moreover, the pin-hole assembly involves delicate maneuvers. Therefore, this task serves to evaluate the performance of the mouth and tongue interactive device in controlling the WRL.

Ten participants are organized to complete the pin-hole assembly task by controlling the WRL through the interactive device. Each participant attempts the task three times. These ten participants are healthy males aged between 20 and 30, and all are first-time users of the experimental equipment. The participants’ heights ranged from 170 cm to 178 cm, and their weights ranged from 75 kg to 85 kg. None of the participants reported a smoking preference, and they were in good health throughout the experiment, without any illness. Throughout the experiment, data acquisition encompasses recording the raw and processed sensor data from the device, three-dimensional motion data of the WRL, one-dimensional force information, the solenoid valve’s on-off status, and the task completion time. After the experiment, each participant is interviewed to gather feedback on their physical and psychological experiences while using the device. These evaluations are asked to include physical load, cognitive load, comfort, and effects on WRL control. Additionally, a fatigue rating survey about each experiment was completed, assessing tongue movement, inhalation, and exhalation. Ratings for each type of operation ranged from 1 to 5, with 5 indicating the highest level of fatigue.

**Figure 4:**
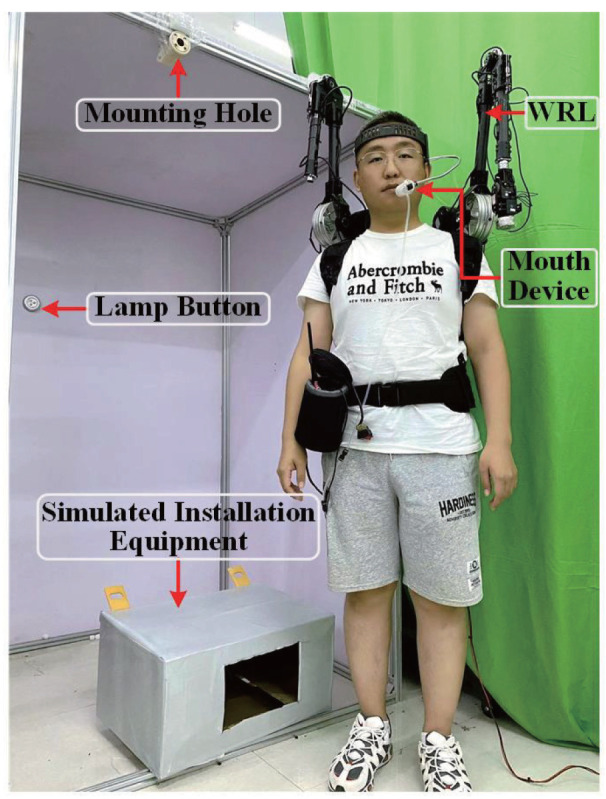
Experimental setup diagram: This figure visually represents the experimental configuration, depicting key components within the task scenario, such as the participant, WRL, the mouth and tongue interactive device, experimental equipment, and other pertinent elements.

The experiment involves the operator completing a pin-hole assembly task during equipment installation. After the operator aligned the device’s hole positions, the WRL executed the latching operation. The experimental setup consists of components, prominently including the mouth and tongue interactive device, the WRL, and simulation equipment, as depicted in Figure 4. The WRL designed in our previous research comprises two operable wearable robotic arms [6]. This study involves the use of only one robotic arm to accomplish the task. The participants, wearing the interactive device affixed to their head, also wear the WRL as part of the experiment setup. The simulated device with mounting holes is secured on a stand, indicating the completion of the task.

**Figure 5:**
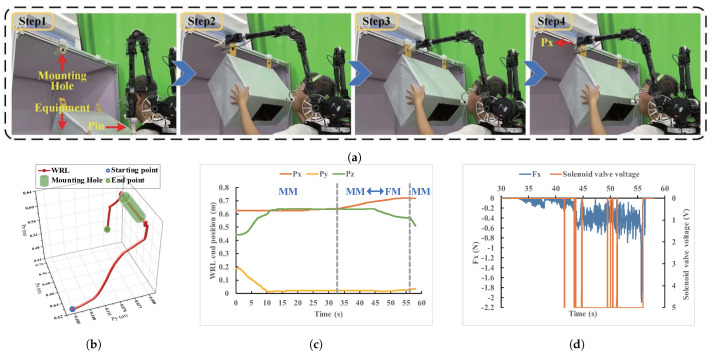
Experiment process diagram (pin-hole assembly task). (**a**) The task can be divided into four sequential steps. (**b**) The three-dimensional motion trajectory of the WRL end effector. (**c**) Detailed position curve of the WRL end effector. (**d**) Curve depicting the variation in the X-directional contact force at the WRL end effector and the corresponding solenoid valve states.

The intricate operational procedure for the pin-hole assembly task is elucidated in Figure 5a. A magnetic pin is affixed to the WRL and can be attached and detached. In Step 1, the operator initiates the task by aligning the holes on the equipment with the installation locations. The control mode is MM. In Step 2, the operator employs the interactive device to preliminary adjust the pin’s alignment to the hole. In Step 3, the control mode enters the FM, and the solenoid valve status can give the operator feedback on the pin-hole contact status. Notably, variations in mouth gas pressure provide a perceptible indicator of pin-hole collisions, allowing the operator to fine-tune the WRL to accomplish the installation. In Step 4, post-installation, the operator controls the movement of the WRL’s end to disengage the pin. The control mode reverts to MM, enabling the WRL to return to its predetermined position.

The system performance of the interactive device will be assessed through the experimental data and subjective evaluations from the participants.

### 4.2. Performance Evaluation

**Figure 6:**
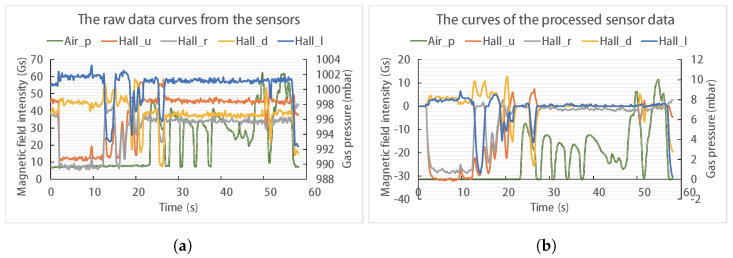
The data curves of internal sensors within the device. (**a**) The raw data curves from the sensors. (**b**) The curves of the processed sensor data.

Every task operation by all participants is successfully completed on the first attempt. During the experimental process, data were collected, including sensor data from the device, WRL data, and task completion times. One participant’s data has been meticulously organized and temporally synchronized, corresponding to two operational modes: MM and FM. Figure 5b shows the spatial trajectory of the WRL end effector, aligned with different task phases. Figure 5c presents the detailed position data for the WRL, along with mode segmentation. In Figure 5d, variations in the X-directional contact force during the operation are depicted, along with the status of the solenoid valve. The solenoid valve’s voltage value directly signifies its operational state, with 5 V indicative of the closed state $V_{c}$ and 0 V indicating the open state $V_{o}$. Figure 6 depicts the Hall sensors and pressure sensor data curves within the device. Due to the presence of noise and drift in the sensors, data processing was conducted. Figure 6a,b represent the raw and processed data curves, respectively. It can be observed that the processed curves are smoother. The processed data serve as the input signal from the participant, forming a correspondence with the motion output of the WRL.

Figure 5d illustrates the data curve derived from the pin-hole assembly task experiment, characterized by a progression from the initial coarse alignment to subsequent fine adjustment. Throughout the preliminary alignment phase, force sensor data register at 0 N, while the control mode remains firmly within MM. The WRL’s movement exhibits a consistent and smooth trajectory. From 33.5 s onward, a slight increase in contact force is observed, indicating that the pin has come into contact with the surrounding hole. Upon reaching a contact force greater than −0.2 N, the solenoid valve transitions into $V_{c}$, signaling the operator that the WRL has made contact with the hole’s vicinity. Consequently, the control mode shifts to FM, initiating fine-tuning. Between 33.5 s and 56 s, the operator continuously adjusts the pin’s installation status, accompanied by real-time contact force feedback from the solenoid valve. Upon successful installation, the operator controls the WRL end effector’s lateral movement to disengage the pin’s magnetic part. A peak contact force of −2.17 N occurred during the operation, likely resulting from an impact at the end of the installation. During the adjustment process by the operator, the contact force stabilized at around −0.4 N, influenced by assembly precision and friction. This indicates that the interactive device enables smooth control of both position and force for the WRL. The real-time response of the solenoid valve played a role in providing some feedback on the contact status.

**Figure 7:**
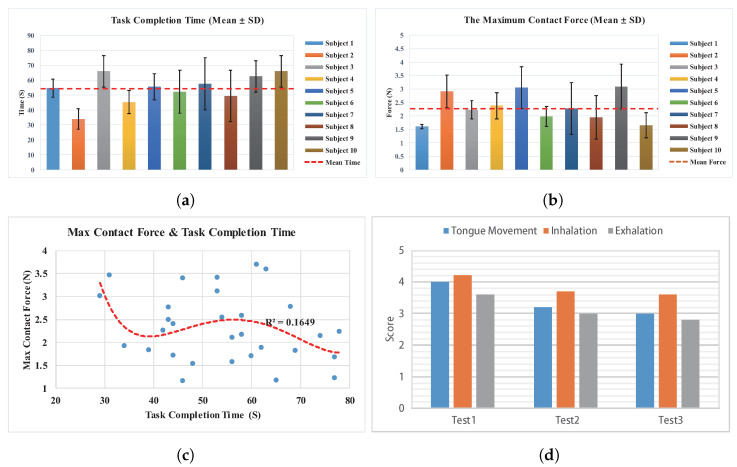
Graphical representation of experimental results for the ten participants. (**a**) Bar chart depicting the mean task completion time for participants. (**b**) Bar chart illustrating the maximum X-direction contact force at the end effector of the WRL during the experimental process. (**c**) Scatter plot depicting the relationship between maximum contact force and task completion time. (**d**) The fatigue rating scores of participants regarding tongue movement, inhalation, and exhalation.

The maximum contact force in the X-direction and the task completion time during the pin-hole assembly operation are statistically analyzed for all participants, as depicted in Figure 7. In the initial analysis, the completion times of participants are examined, as illustrated in Figure 7a. The disparities in task completion times among participants are little, with an average time of 54.4 s. However, one participant exhibits a notably shorter completion time of 34.0 s, potentially attributed to individual differences. The interactive device demonstrates adaptability across diverse individuals, yielding comparable task completion efficiencies.

Subsequently, the peak contact force in the X-direction at the end effector of the WRL during participant operations is analyzed, as shown in Figure 7b. Variability in the manipulation capabilities of participants with the interactive device is observed, with contact force ranging from a minimum of 1.6 N to a maximum of 3.1 N and an average force of 2.3 N. Notably, a reverse proportional relationship between task completion times and peak contact force is evident for some participants. Consequently, a further analysis of the relationship between contact force and operation time is conducted, as depicted in Figure 7c. The results indicate that, although an increase in operation time corresponded to a reduction in contact force in some instances, this trend is not pronounced, and the correlation is insufficient. Thus, the speed at which participants complete the task has little impact on the contact force. The contact force remains constrained within the range of approximately 2.3 N, demonstrating suitability for intricate operational requirements.

A summary of fatigue rating data for participants’ tongue movement, inhalation, and exhalation is provided in Figure 7d. The data indicate that inhalation ranks higher than tongue movement in terms of fatigue severity, which, in turn, is higher than exhalation. This suggests that participants find tongue movement and inhalation more strenuous. The results also demonstrate a decrease in fatigue levels with continued use, with the most significant difference observed between the initial and subsequent uses. This could be attributed to participants’ increasing proficiency with the task, indicating the potential for fatigue reduction through training. A summary of the subjective evaluation of all participants is then compiled. All participants express curiosity about the use of the interactive device and looked forward to using it to control the WRL. Some negative evaluations include the device placing somewhat of a burden on the tongue, leading to fatigue. Additionally, the device has an impact on the natural breathing of users, making it challenging to achieve long-term continuous control through breathing in and out. Positive evaluations include the perception that the interactive device is direct and easy to use for eight participants. Seven participants regard this device’s control over the WRL as flexible, enabling complex operations. The changes in mouth airflow resistance are noticeable, allowing them to perceive the contact status of the WRL end effector.

## 5. Discussion

This study presents an innovative method for human–robot interaction with WRLs by utilizing a mouth and tongue interface, enabling motion control and feedback. The interactive device distinguishes itself with its compact design, allowing users to manipulate and control WRL with precision and simplicity. However, limitations include the burden on the operator’s tongue and the potential impact on breathing. The burden on the tongue and the respiratory impact of the device can be addressed through training to improve operator proficiency, thereby reducing the device’s adverse effects. Additionally, fatigue resulting from tongue movement can be mitigated by improving the rubber plug to a resetting mechanism and reducing the damping of the controller. Similarly, reducing the device’s ventilation ports to minimize the required gas volume can alleviate fatigue resulting from breathing. Subsequent research will focus on optimizing the device’s performance to enhance tongue comfort and minimize the impact on breathing.

The interactive device includes a feedback mechanism that controls the on–off state of the solenoid valve. Although this feedback mechanism primarily provides binary information to indicate contact or non-contact, it has demonstrated effectiveness in experimental settings. By utilizing collision feedback, operators can achieve precise control in tasks such as pin-hole assembly. Our main objective is to obtain more comprehensive feedback regarding the interaction between the WRL and the environment, leveraging the inherent sensitivity of the tongue and mouth. Achieving this goal may require integrating multimodal feedback sensors and strategies.

## 6. Conclusions

This paper presents a novel concept for manipulating WRL using an interactive device operated by the mouth and tongue. The proposed device establishes a bidirectional communication channel to control and sense the WRL, particularly in scenarios where human limbs are occupied. Two distinct control modes, namely the movement mode and force-position hybrid mode, have been developed. Using the device, the operator can control the two degrees of freedom motion of the WRL by manipulating a tongue-based controller. Additionally, the mouth’s blowing and inhaling actions enable one-dimensional movement and control of contact force. Sensing of contact feedback from the WRL is achieved through mouth pressure. To evaluate the performance of this interactive device, ten participants have been organized to conduct pin-hole assembly experiments. The results demonstrate that participants are able to achieve precise control of position and force in the WRL, while also perceiving feedback. The device proved to be user-friendly and versatile; although, limitations were observed in terms of comfort and its impact on breathing. However, these limitations can be mitigated through training or device design improvements. This study demonstrates the capability of tongue and mouth sensors to achieve WRL operation and feedback, as well as their potential applications in industrial settings.

## Data Availability

Data are contained within the article.

## Abbreviations

The following abbreviations are used in this manuscript:

WRL	Wearable Robotic Limb
EMG	Electromyography
EEG	Electroencephalography
MM	Movement Mode
FM	Force-Position Hybrid Mode
IMUs	Inertial Measurement Units
